# Modification of Hinge/Transmembrane and Signal Transduction Domains Improves the Expression and Signaling Threshold of GXMR-CAR Specific to *Cryptococcus* spp.

**DOI:** 10.3390/cells11213386

**Published:** 2022-10-26

**Authors:** Matheus Henrique dos Santos, Michele Procópio Machado, Pappanaicken R. Kumaresan, Thiago Aparecido da Silva

**Affiliations:** 1 Department of Cell and Molecular Biology and Pathogenic Bioagents, Ribeirão Preto Medical School, University of São Paulo, Ribeirão Preto 140490-900, SP, Brazil; 2 Department of Hematopoietic Biology and Malignancy, The University of Texas MD Anderson Cancer Center, Houston, TX 77030, USA

**Keywords:** chimeric antigen receptor, GXMR-CAR, *Cryptococcus* spp., invasive fungal infection

## Abstract

Chimeric antigen receptors (CARs) redirect T cells to recognize a specific target. CAR components play a pivotal role in antigen specificity, structure stability, expression on cell surface, and induction of cellular activation, which together determine the success of CAR T-cell therapy. CAR products targeting B-cell lymphoma encouraged the development of new CAR applications beyond cancer. For example, our group developed a CAR to specifically target glucuronoxylomannan (GXM) in the capsule of *Cryptococcus* species, called GXMR-CAR or GXMR-IgG4-28ζ. *Cryptococcus* are fungi that cause the life-threatening disease cryptococcosis, and GXMR-IgG4-28ζ redirected T cells to target yeast and titan cell forms of *Cryptococcus* spp. Here, we replaced the IgG4-hinge and CD28-transmembrane domains from GXMR-CAR with a CD8α molecule as the hinge/transmembrane and used CD28 or 4-1BB molecules as co-stimulatory domains, creating GXMR-8-28ζ and GXMR-8-BBζ, respectively. Jurkat cells expressing GXMR-CAR containing CD8α as the hinge/transmembrane improved the CAR expression and induced a tonic signaling. GXMR-8-28ζ and GXMR-8-BBζ induced high levels of IL-2 and up-regulation of CD69 expression in the presence of reference strains of *C. neoformans* and *C. gattii*. Moreover, GXMR-8-28ζ and GXMR-8-BBζ showed increased strength in response to incubation with clinical isolates of *Cryptococcuss* spp., and 4-1BB co-stimulatory domain triggered a more pronounced cellular activation. Dasatinib, a tyrosine kinase inhibitor, attenuated the GXMR-CAR signaling cascade’s engagement in the presence or absence of its ligand. This study optimized novel second-generation GXMR-CARs containing the CD8-hinge/transmembrane domain that improved CAR expression, antigen recognition, and signal strength in T-cell activation.

## 1. Introduction

Chimeric antigen receptors (CARs) are recombinant receptors with the capacity to target any molecule by an antigen recognition domain that is commonly composed of a single-chain variable fragment (scFv) derived from a monoclonal antibody [1]. A CAR’s non-antigen-binding extracellular region is composed of a hinge/spacer portion; immunoglobulin Fc, CD8α, or CD28 molecules are commonly used for this purpose [2]. A CAR’s transmembrane domain provides stability to the receptor and is available to associate with other cell surface proteins [3]; sequences derived from CD8α and CD28 molecules are often adopted as transmembrane domains [4]. Signal transduction through the CAR receptor is guaranteed by fusion of the CD3ζ cytoplasmic domain with a co-stimulatory domain, such as CD28 or 4-1BB, which characterizes a second-generation CAR design [4].

The application of CAR T cells in immunotherapy for leukemia and lymphoma has revolutionized adoptive cell therapy. In 2017, the US Food and Drug Administration approved the first CAR T-cell product manufactured to target B-cell lymphoma [5]. Major successes in oncology have led to many possibilities of using CARs to redirect immune cells to treat autoimmune diseases [6] and infections caused by viruses or fungi [7]. The importance of T cells and natural killer (NK) cells in antifungal immunity is well established [8,9], and previous studies have demonstrated the capacity of CAR-modified cells to target invasive forms of *Aspergillus fumigatus* and *Cryptococcus* spp. [10,11,12]. Therefore, there is a growing scientific interest in the use of CARs against invasive fungal infections.

Among the fungal species responsible for invasive infections worldwide, *Cryptococcus gattii* and *Cryptococcus neoformans* present a serious global health threat, with 220,000 cases of cryptococcosis and 180,000 related deaths reported annually [13]. Propagules or yeasts of *Cryptococcus* spp. reach the alveolar space after inhalation, culminating in pulmonary tissue infection, which is a predilection site for *C. gattii*, whereas *C. neoformans* has a predilection to disseminate to the central nervous system, where it causes cryptococcal meningitis [14]. Immunocompromised patients, such as those with HIV/AIDS, are mainly infected with *C. neoformans*, whereas the rate of immunocompetent individuals infected with *C. gattii* is greater [15,16]. The host immune response against *Cryptococcus* spp. is impaired by the polysaccharide capsule of yeast, which is considered to be the major virulence factor that subverts the development of adaptive immune response [17]. Glucuronoxylomannan (GXM) represents more than 90% of the capsule, and GXM’s structure is highly influenced by the microenvironment to confer resistance against oxygen and nitrogen reactive species in a host–pathogen interaction [18]. Moreover, the mannosyl residues in GXM can be acetylated or replaced by other sugar moieties; therefore, the GXM structure varies between *Cryptococcus* species, which is a factor that compromises the phagocytic activity of antigen-presenting cells [19,20]. Unfortunately, the control of cryptococcosis progression is limited to the use of azole, polyene, and pyrimidine analogs, which are associated with adverse effects and increased resistance to antifungal drugs in *Cryptococcus* spp. [21,22].

A promising strategy to treat fungal infections is to use adoptive T-cell therapies, which have the potential to be translated from bench to bedside [23]. The first attempt to engineer CAR T cells against invasive fungal infections considered the carbohydrate recognition domain of the Dectin-1 receptor, which has specificity to β-glucans and makes up the antigen recognition portion of a CAR to target the cell wall of *Aspergillus fumigatus* conidia [10]. The Dectin-1 CAR’s construction and its expression by T cells allowed the control of conidium-hypha switching in *A. fumigatus*, compromising the progression of experimental aspergillosis [10]. A second CAR specific to invasive fungal infections was developed by our group to target GXM polysaccharides on the *Cryptococcus* spp. capsule using an antigen recognition domain derived from an anti-GXM monoclonal antibody (18B7 clone [24]) [11]. This novel second-generation CAR was designated GXMR-CAR and is composed of an IgG4 Fc-derived hinge, CD28 transmembrane and co-stimulatory signal transducer, and CD3ζ. T cells expressing GXMR-CAR, when infused in mice infected with *C. neoformans*, reduced the frequency of titan cells in the pulmonary tissue [11], and GXMR-CAR redirected T cells to recognize yeast and titan cell forms of *C. gattii* and *C. neoformans* [12].

The threshold in CAR T-cell activation can be affected by all portions of the CAR, and often the hinge/transmembrane (derived from IgG4-hinge or CD8α molecules) and intracellular domains (derived from CD28 or 4-1BB molecules) play a pivotal role in CAR expression and cell activation, which impacts the final effect of CAR T cells against the target [25]. The purpose of the present study was to optimize second-generation GXMR-CARs to more effectively mediate the cell activation against *Cryptococcus* spp. Toward this aim, we generated novel GXMR-CARs by replacing the IgG4 hinge and CD28 transmembrane with CD8 hinge/transmembrane, with CD28 or 4-1BB used as co-stimulatory domains followed by the CD3ζ cytoplasmic domain. The original forms of GXMR-CAR (GXMR-IgG4-28ζ) and GXMR-CAR-modified constructs (GXMR-8-28ζ and GXMR-8-BBζ) were expressed in Jurkat cells after lentiviral transduction. The capacity of distinct GXMR-CAR constructs to mediate the activation of engineered Jurkat cells in the presence of *C. gattii* or *C. neoformans* reference strains and clinical isolates was compared between GXMR-CAR constructs. In the GXMR-CAR-modified constructs, the CD8α molecules as hinges/transmembranes improved CAR expression and mediated strong T-cell activation. Moreover, 4-1BB, as a co-stimulatory domain, triggered a signal strength that was more pronounced in response to *Cryptococcus* spp. Further, dasatinib, an inhibitor of Src family kinases, attenuated GXMR-CAR signaling, indicating that Src family kinases are crucial in this pathway. These results were obtained using engineered Jurkat cells that demonstrated a feasible workflow to evaluate the signaling threshold of novel CAR constructs specific to *Cryptococcus* spp.

## 2. Materials and Methods

### 2.1. Cell Lines and Growth Conditions

Jurkat (clone E6-1) and HEK-293T cell lines were purchased from the Rio de Janeiro Cell Bank (BCRJ). Jurkat cells were cultured in Roswell Park Memorial Institute (RPMI) medium (Thermo Fisher Scientific, Waltham, MA, USA) supplemented with 10% fetal bovine serum (Gibco, Waltham, MA, USA), 1% ampicillin/streptomycin (Sigma-Aldrich, St. Louis, MO, USA; cat. P4333), and 100 mM of sodium pyruvate (Gibco, Waltham, MA, USA). HEK-293T cells were cultivated in high-glucose Dulbecco’s modified Eagle’s medium (Corning, NY, USA) supplemented with 10% fetal bovine serum, 1% ampicillin/streptomycin, and 100 mM of sodium pyruvate. Cells were maintained in a humid atmosphere with 5% CO_2_ at 37 °C and sub-cultured every 2–3 days.

### 2.2. Culture of Cryptococcus *spp*.

The *Cryptococcus* species *C. gattii* R265 (VGII molecular genotype) and *C. neoformans* H99 (serotype A) were grown overnight in yeast nitrogen base (YNB) (Sigma) medium at 30 °C with vigorous shaking (150 rpm) for 20 h. The cells were harvested, washed twice with phosphate buffered saline (PBS), and resuspended in PBS to perform heat inactivation (70 °C for 50 min) of the yeast cells, which were stored at −80 °C in a 25% glycerol/PBS solution. Before the experiment, an aliquot of heat-inactivated yeast was thawed and washed twice in PBS to determine cell concentration using a Neubauer chamber. The same procedures described above were performed using clinical isolates of *C. gattii* and *C. neoformans* provided by Prof. Dr. Roberto Martinez, Ribeirão Preto Medical School, University of São Paulo, Brazil. The clinical isolates of *C. neoformans* (455 and 478) and *C. gattii* (521, 523, and 525) were obtained from cerebrospinal fluid (CSF), and clinical isolate of *C. neoformans* (474) was obtained from bronchoalveolar lavage (BAL).

### 2.3. Construction of Plasmids Encoding Anti-GXM CARs to Target Cryptococcus *spp*.

GXMR-CAR was designed to contain a variable region sequence of a full mouse antibody (18B7 clone) with specificity to the capsular component GXM, as reported by Casadeval et al. [24]. Previously, GXMR-CAR was generated by linking the binder sequence (single chain variable fragment specific to GXM) to the IgG4 hinge and CD28 transmembrane domain (UniProt P10747, 153–179 aa position) and then to CD28 (UniProt P10747, 180–220 aa position) and CD3ζ (UniProt P20963, 52–164 aa position) as signaling domains, as reported by da Silva et al. [11]. In the current study, two variants of GXMR-CAR were designed using a GXM-binding domain sequence derived from anti-GXM 18B7 monoclonal antibody [24] with the following components from 5′ to 3′: the hinge and transmembrane regions of the human CD8α molecule (UniProt P01732, 136–206 aa position), the cytoplasmic portion of the human CD28 (UniProt P10747, 180–220 aa position) or 4-1BB (UniProt Q07011, 214–255 aa position) molecules, and the cytoplasmic region of the human CD3ζeta molecule (UniProt P20963, 52–164 aa position). These CARs were designated as GXMR-8-CD28ζ and GXMR-8-BBζ. CAR-encoding sequences were subcloned into a second-generation lentiviral plasmid backbone (pLentiCas9-EGFP, GenScript, NJ, USA) containing the GFP sequence as a reporter, and the plasmid backbone containing the GFP sequence alone was considered as a control lentiviral (Lenti-mock) vector. GXMR-8-CD28ζ and GXMR-8-BBζ were sequenced and verified at the Sequencing Facility of the School of Pharmaceutical Sciences of Ribeirao Preto, University of São Paulo.

### 2.4. Production of Lentiviral Particles Containing a GXMR-CAR Sequence

Lentiviral particles containing the plasmid encoding the GXMR-CAR construct were produced in the HEK-293T cell line grown as described above. The cells were seeded in 25 cm^3^ cell culture flasks at a concentration of 2.5 × 10^6^ cells/5 mL of fresh medium; after 48 h of culture, the supernatant volume was reduced to 3 mL before transfection. The plasmid pMD2.G (VSV-G envelope-expressing plasmid cat. no. 12259; Addgene, Watertown, MA, USA) and psPAX2 ((second-generation lentiviral packaging plasmid cat. no. 12260; Addgene, Watertown, MA, USA) packaging plasmids were co-transfected with the GXMR-CAR lentiviral vector using Lipofectamine 3000 (Thermo Fisher, cat. L3000001) transfection reagent according to the manufacturer’s instructions. Twenty-four hours after transfection, 2 mL of fresh medium was added to the culture, and the supernatant-containing lentiviral particles were collected 48 and 72 h after transfection. The lentiviral particles were cleared by centrifugation (300× *g*, 10 min, 4 °C), and the supernatant was incubated with Lenti-X concentrator (cat. 631232; Takara Bio USA, Inc., Mountain View, CA, USA) to concentrate the lentiviral stock following the manufacturer’s instructions. The lentiviral stock was resuspended in PBS, and aliquots were stored at −80 °C until use.

Lentiviral transduction of the Jurkat cell line was used to measure the lentiviral particle titer, and a spinoculation protocol (850× *g*, 65 min, RT) was used for transduction. Cells were seeded in 48-well plates at concentration of 1 × 10^5^ cells/250 µL, and 72 h after transduction, the percentage of Jurkat cells expressing the vector-encoded gene product was represented by GFP expression as determined by flow cytometry. The titer was calculated based on the dilution of lentiviral particles that resulted in GFP expression between 10% and 30%, and the following formula was used: titer = {[(%GFP/100) × dilution × cell seeded]/final volume}.

### 2.5. Transduction of Jurkat Cells

The transduction of Jurkat cells was performed using a spinoculation protocol (850× *g*, 65 min, RT) with MOIs of 1 and 3 of lentiviral particles of GXMR-IgG4-CD28ζ, GXMR-8-CD28ζ, GXMR-8-BBζ, and Lenti-mock. Jurkat cells were plated on 48-well plates at a concentration of 1 × 10^5^ cells/250 µL, and lentiviral particles were added before the spinoculation. Seventy-two hours after transduction, GFP expression was measured using flow cytometry (Guava EasyCyte, Guava Technologies, Millipore, Burlington, MA, USA) to determine the transduction efficiency. The cells were either used for the experiments or maintained in culture at a concentration of 3–5 × 10^5^ cells/mL.

### 2.6. Detection of GXMR-CAR on the Cell Surface

The expression of GXMR-CAR on the cell surface was detected as previously reported by da Silva et al. [11], in which modified cells were incubated with soluble GXM extracted from *C. gattii* [26], and the interaction between GXMR-CAR and its ligand was revealed by anti-GXM, clone 18B7, which is a mouse monoclonal antibody (Sigma; cat. MABF2069). These procedures were performed using 1 × 10^6^ GXMR-CAR Jurkat cells incubated with GXM (200 µg/mL) for 40 min at 4 °C. The cells were then washed with PBS and incubated with anti-GXM monoclonal antibody for 40 min at 4 °C. The cells were washed with PBS and incubated with biotin-conjugated secondary antibody for 45 min at 4 °C, followed by washing and incubation with PE-conjugated streptavidin for 45 min at 4 °C. Non-transduced Jurkat cells were subjected to the same procedures described above and considered as negative controls for GXM binding. The detection of GXMR-CAR on the cell surface was evaluated using flow cytometry (Guava EasyCyte, Guava Technologies, Millipore), and the data obtained were analyzed using FlowJo™ software (version 10, for Windows; Becton, Dickinson and Company, Ashland, OR, USA; 2019).

### 2.7. GXMR-CAR-Jurkat Cell Activation Assay

Transduced Jurkat cells at an MOI of 1 and 3 to express distinct variants of GXMR-CAR were plated in 96-well plates at a concentration of 2 × 10^5^ cells/200 µL and incubated with soluble GXM (0.1, 1, or 10 µg/mL) or heat-killed yeast of *C. gattii* or *C. neoformans* using 1:1 ratio (cells:yeast). After 24 and 48 h of incubation, the concentration of viable GXMR-CAR Jurkat cells was determined using flow cytometry and propidium iodide staining. The production of IL-2 by the cells was considered as indicative of cell activation, and the cell supernatant was collected to measure the cytokine production using the BD OptiEIA Human IL-2 ELISA kit (BD Biosciences, San Jose, CA, USA), according to the manufacturer’s instructions. Jurkat cells transduced with mock-GFP lentiviral particles were used as a negative control for cell activation.

The expression of the activation marker CD69 on the cell surface of transduced Jurkat cells at an MOI 3 was evaluated after co-culture with heat-killed yeast of *C. gattii* and *C. neoformans*. After 20 h of incubation, Jurkat cells modified with GXMR-CAR constructs were washed with PBS and incubated with APC-conjugated anti-human CD69 antibody for 40 min (ThermoFisher, cat. 17-0699-42). The cells were washed with PBS, the CD69 expression was measured by flow cytometry BD LSRFortessa (BD Bioscences, San Jose, CA, USA), and the values were expressed as median fluorescence intensity (MFI).

### 2.8. Evaluation of Tyrosine Kinase Inhibitor (Dasatinib) and Phosphatase CD45 Inhibitor (NQ301) on GXMR-CAR-Jurkat Cells

MOI 1 and MOI 3 GXMR-CAR-transduced Jurkat cells were seeded in 96-well plates at a concentration of 2 × 10^5^ cells/mL in 200 µL of RPMI medium. Various concentrations of dasatinib or NQ301 were added to the cells, and the same concentration of DMSO was used as a control. The cells were incubated for 24 and 48 h, and the supernatant was collected to assess IL-2 production by the cells. In addition, the effect of dasatinib was evaluated in cells incubated with heat-killed yeast of *C. gattii* or *C. neoformans.* For this, the cells were seeded at the same concentration of 2 × 10^5^ cells/mL in 200 µL of RPMI medium, and 50 nM of dasatinib was added. After 3 h, heat-killed yeasts were added at a cell:yeast ratio of 1:1, and the co-culture was incubated for 24 and 48 h. The cell supernatant was collected to assess IL-2 production, and cell expansion was evaluated under these conditions.

### 2.9. Statistical Analysis

Data were analyzed using Prism 9.0 (GraphPad Software). The normality of all statistical determinations was analyzed using the Shapiro–Wilk test. The homogeneity of variances was analyzed using Bartlett’s test for three or more groups. Analysis of variance (ANOVA) was applied to experiments with three or more groups when the samples had Gaussian distributions. For datasets with a non-normal distribution, the Kruskal–Wallis test was used for experiments with three or more groups. Differences between the means of groups were evaluated by one-way ANOVA followed by Tukey’s multiple comparisons test or Kruskal–Wallis test followed by Dunn’s multiple comparisons test. Differences were considered statistically significant at *p* < 0.05. Results are presented as mean ± standard deviation (SD) or median and interquartile range.

## 3. Results

### 3.1. GXMR-CAR Expression Is Improved by Using CD8α Molecule as Hinge/Transmembrane Domain

**Figure 1:**
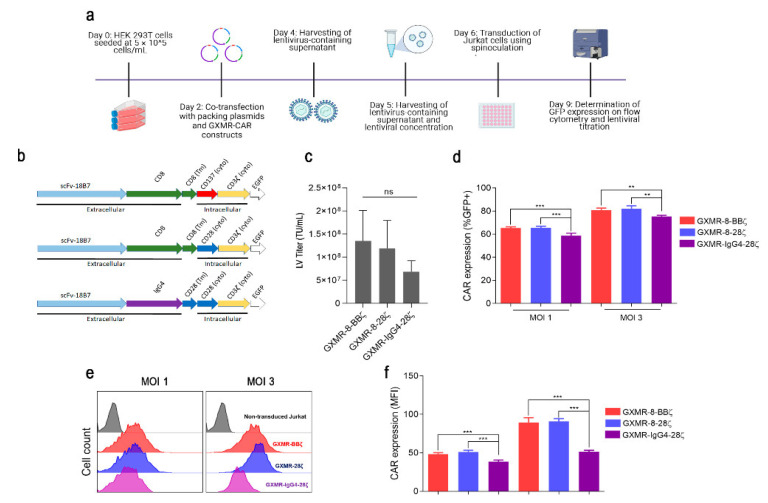
GXMR-CAR expression in Jurkat cells is enhanced by CD8α molecule as hinge/transmembrane domain. Schematic protocol for production of lentiviral particles carrying the GXMR-CAR sequence, the steps in the transfection of HEK 293-FT cells for GXMR-CAR lentiviral packaging, and the procedure to calculate the titer of lentiviral particles harvested and concentrated (**a**). DNA sequence for each GXMR-CAR construct indicating the recognition portion of GXM (scFv obtained from anti-GXM antibody, clone 18B7) that is similar for all constructs, and hinge/transmembrane and intracellular domains as specified in the scheme (**b**). Lentiviral particle titer of distinct GXMR-CAR constructs representative of five independent batches (**c**). Jurkat cells were transduced with GXMR-CAR at an MOI of 1 and 3, and after 3 days, the cells were analyzed using flow cytometry to measure the percentage of GXMR-CAR-positive cells (**d**) and the expression of GXMR-CAR (**e**,**f**). Data are expressed as mean ± SD. Each experiment was performed in 3 to 5 replicates and repeated at least three times. ** *p* < 0.01, *** *p* < 0.001, according to one-way ANOVA. ns, not significant.

Previous studies have demonstrated that GXMR-CAR-IgG4-CD28ζ redirects Jurkat cells to bind to titan cells and yeast forms of *C. gattii* and *C. neoformans*, and soluble GXM extracted from *C. gattii* and *C. neoformans* is strongly recognized by GXMR-CAR-IgG4-CD28ζ [11,12]. Seeking to improve the original form of GXMR-CAR, we initially replaced the hinge/transmembrane domain with the CD8 molecule (Figure 1b). This modification to the GXMR-CAR was followed by the insertion of distinct signal transduction domains that originated from GXMR-CD8α-CD28-CD3ζ (GXMR-8-28ζ) and GXMR-CD8α-4-1BB-CD3ζ (GXMR-8-BBζ), which contain CD28 and 4-1BB, respectively, as co-stimulatory molecules (Figure 1b). In this scenario, the impact of the hinge/transmembrane and intracellular regions in the modified GXMR-CAR was verified according to their transduction efficiency, cell expansion, and cell activation compared to the original form of GXMR-CAR. Jurkat cells were transduced with a multiplicity of infection (MOI) of one or three using lentiviral particles carrying a DNA sequence specific to the GXMR-CAR constructs cited above. The production of lentiviral particles is described in detail in the Materials and Methods section, and the schematic protocol in Figure 1a represents the 9-day workflow starting with the transfection of HEK-293T cells and ending with the lentivirus titer performed on Jurkat cells. The measurement of the lentivirus titer demonstrated a slight difference among GXMR-CAR constructs, which was not significant, and the transduction units per mL (TU/mL) were maintained between 10^7^ and 10^8^ (Figure 1c). The transduction efficiency of GXMR-CAR constructs and their expression levels were determined by the percentage of green fluorescent protein (GFP)-positive cells and median fluorescence intensity (MFI), respectively. Transduction of Jurkat cells was performed using MOIs of one and three, and as expected, the transduction efficiency of GXMR-CAR constructs was higher at an MOI of three (Figure 1d). The percentage of GFP-positive cells was significantly higher in Jurkat cells expressing GXMR-CAR containing CD8α-hinge/transmembrane than in cells modified with GXMR-IgG4-28ζ (Figure 1d). These results were observed for both MOI of one and three, which also demonstrated that the MFI of GXMR-8-28ζ and GXMR-8-BBζ expression was substantially higher than that of GXMR-IgG4-28ζ (Figure 1e,f). Taken together, these results showed that the CD8α molecule in the hinge/transmembrane of GXMR-CAR is accounted for the improvement of the transduction efficiency of GXMR-CAR in Jurkat T cells.

### 3.2. GXMR-8-BBζ Is Highly Expressed during Jurkat Expansion

**Figure 2:**
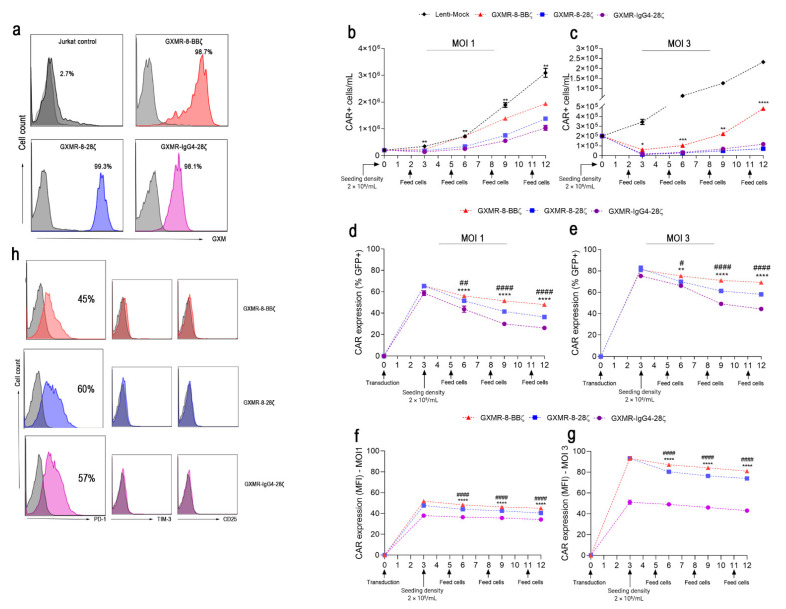
GXMR-8-BBζ expression stands out over GXMR-8-28ζ and GXMR-IgG4-28ζ during Jurkat expansion. Jurkat cells expressing GXMR-CAR were incubated with soluble GXM from *C. gattii* R265 (*n* = 3 per condition), as described in the Materials and Methods section (**a**). The representative histograms show the percentage of GXMR-CAR Jurkat cells able to recognize GXM on the cell surface, and the fluorescence intensity (*x*-axis, MFI) in the histogram represents the labelled anti-GXM antibody that interacted with soluble GXM recognized by GXMR-CAR Jurkat cells. Jurkat cells were transduced with MOI 1 (**b**,**d**,**f**) or MOI 3 (**c**,**e**,**g**) GXMR-CAR construct or Lenti-mock, and the cell concentration (cells/mL; **b**,**c**), percentage of CAR-positive cells (**d**,**e**), and CAR expression (represented by MFI; **f**,**g**) were measured using flow cytometry to detect GFP expression. These parameters were evaluated within 12 days of culture; * denotes statistical significance between GXMR-8-BBζ and GXMR-IgG4-28ζ, and # denotes statistical significance between GXMR-8-28ζ and GXMR-8-BBζ compared to GXMR-IgG4-28ζ. GXMR-Jurkat cells were evaluated after exclusion of dead cells using a propidium iodide marker (**b**–**g**). Expression of exhaustion markers, PD-1 and TIM-3, and an activation marker, CD25, on day 4 after transduction of Jurkat cells using distinct GXMR-CAR constructs (**h**). The representative histograms show the percentage of positive cells within CAR+ cells gated. The values are expressed as mean ± SD, and scatter plots represent triplicates. #, * *p* < 0.05; ##, ** *p* < 0.01; *** *p* < 0.001; ####, **** *p* < 0.0001, according to one-way ANOVA.

When GXMR-CAR constructs were expressed in Jurkat cells, the presence of GXMR-CARs on the cell surface was investigated by evaluating the recognition of soluble GXM (extracted from *C. gattii* R265) by GXMR-CAR, as previously reported [12]. In Figure 2a, soluble GXM was recognized by GXMR-CAR Jurkat cells, whereas the control cells were unable to interact with soluble GXM. To evaluate the effect of GXMR-CAR expression on Jurkat cell proliferation, transduction at MOIs of one and three was performed using GXMR-IgG4-28ζ, GXMR-8-28ζ, or GXMR-8-BBζ constructs. Jurkat cells were also transduced with a lentiviral backbone vector (Lenti-mock) carrying the GFP marker only, and the growth curve and percentage (among GFP-positive cells) of CAR-expressing cells were measured every 3 days for 12 days (Figure 2b–e). As shown in Figure 2b,c (MOI 1 and MOI 3, respectively), the cellular expansion of Jurkat cells expressing GXMR-CAR was significantly reduced compared to Lenti-mock, and only transduction with an MOI of three demonstrated a greater expansion of GXMR-8-BBζ Jurkat cells compared to GXMR-8-28ζ and GXMR-IgG4-28ζ (Figure 2d). Moreover, the percentage of Jurkat cells expressing GXMR-8-BBζ showed better maintenance in all periods analyzed, and GXMR-CARs containing a CD8α-hinge/transmembrane allowed a higher percentage of GFP-positive cells in comparison to GXMR-IgG4-28ζ Jurkat cells over time during expansion (Figure 2d,e). Similarly, GXMR-8-BBζ Jurkat cells maintained a higher intensity of CAR expression over all periods, and GXMR-8-28ζ expression was lower than that in GXMR-8-BBζ cells (Figure 2f,g). GXMR-IgG4-28ζ Jurkat cells had the lowest CAR expression intensity compared to the other constructs after transduction at an MOI of three (Figure 2f,g). Considering the effect of GXMR-CARs on Jurkat cell expansion, the expression of exhaustion markers PD-1 and TIM-3 was measured in Jurkat cells modified with GXMR-CAR constructs at an MOI of three. Only PD-1 expression was augmented in Jurkat cells expressing GXMR-CARs, without a significant difference between them, whereas TIM-3 expression was not detected (Figure 2h). In addition, the presence of the activation marker CD25 was assayed in GXMR-CARs Jurkat cells, and CAR expression did not alter the absence of CD25 expression previously reported in Jurkat cells (Figure 2h).

### 3.3. Pivotal Role of CD8α-Hinge/Transmembrane in GXMR-CAR Tonic Signaling That Has Signal Strength Enhanced by Co-Stimulatory Molecule 4-1BB

**Figure 3:**
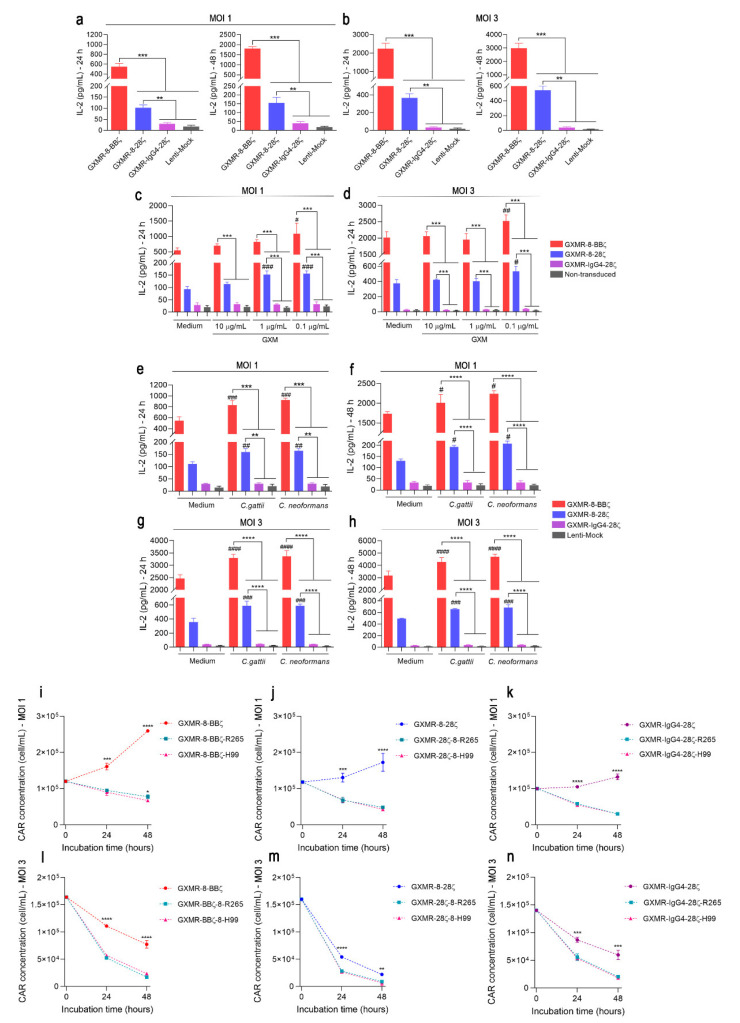
Jurkat cells expressing GXMR-8-BBζ demonstrate a greater signal strength in the presence or absence of *Cryptococcus* spp. Jurkat cells were transduced with distinct GXMR-CAR or Lenti-mock at an MOI of 1 or 3, and on day 3 after transduction, the modified cells (2 × 10^5^ cells/mL) were incubated for 24 and 48 h to evaluate tonic signaling by measuring the production of IL-2 using ELISA (**a**,**b**). In addition, modified Jurkat cells (2 × 10^5^ cells/mL) were incubated with different concentrations of soluble GXM from *C. gattii* R265 for 24 h (**c**,**d**) and also incubated with heat-killed yeast of *C. gattii* R265 or *C. neoformans* H99 at a ratio of 1:1 (cells:yeast) for 24 and 48 h of culture (e-h). After 24 and 48 h of incubation, IL-2 levels in the culture supernatant were quantified using ELISA (**c**–**h**), and cell expansion within 48 h of culture was evaluated using flow cytometry to detect GFP expression, and dead cells were excluded using a propidium iodide marker to determine the cell concentration (cells/mL; **i**–**n**). The values are expressed as mean ± SD. Each experiment was performed in triplicate and repeated at least three times. # *p* < 0.05; **, ## *p* < 0.01; ***, ### *p* < 0.001; ****, #### *p* < 0.0001, according to one-way ANOVA. In (**c**–**h**), # denotes statistical significance compared to medium group, and * represents statistical significance compared to other GXMR-CAR in the same condition. In (**i**–**n**), # represents statistical significance between GXMR-CAR incubated with or without *Cryptococcus* spp.

CAR molecules frequently produce tonic signaling that can be associated with their high surface density and self-aggregation, and CAR tonic signaling could be characterized as “non-coordinated and sustained activation of the T cell in either a ligand-independent or -dependent fashion” [27]. To assess GXMR-CAR tonic signaling, Jurkat cells expressing distinct GXMR-CAR constructs, after transduction with MOI 1 or 3, were cultivated for 24 and 48 h, and the supernatant was used to measure the levels of IL-2 by enzyme-linked immunosorbent assay (ELISA) that represents cell activation. The cells modified with the constructs carrying CD8α as hinge/transmembrane (GXMR-8-BBζ and GXMR-8-28ζ) produced remarkably higher levels of IL-2 than cells transduced with the Lenti-mock construct (Figure 3a,b). These results demonstrate that GXMR-CAR tonic signaling occurs in a hinge/transmembrane-dependent manner, and the levels of IL-2 released by GXMR-8-BBζ and GXMR-8-28ζ Jurkat cells were higher at an MOI of three, which suggests a relationship between GXMR-CAR expression level and the strength of tonic signaling. Moreover, GXMR-8-BBζ, which contains co-stimulatory molecule 4-1BB, induced levels of IL-2 after 48 h incubation that were 11- and 6-fold higher than those in cells expressing GXMR-8-28ζ at an MOI of one and three, respectively (Figure 3a,b). These findings show that GXMR-CAR expressed by Jurkat cells triggers tonic signaling, and the hinge/transmembrane and co-stimulatory domains are crucial in the strength of tonic signaling.

### 3.4. T-Cell Activation Signal Is Strengthened by GXMR-8-BBζ in the Presence of Cryptococcus spp. Yeast

To address the substantial impact of GXMR-CAR tonic signaling on the activation of GXMR-CAR Jurkat cells against its ligand, soluble GXM extracted from *C. gattii* was incubated with MOI 1 and MOI 3 transduced cells, and the levels of IL-2 in the cell supernatant were measured after 24 h of culture. At both MOIs, GXMR-8-BBζ- and GXMR-8-28ζ-modified cells incubated with GXM showed a significant increase in IL-2 levels compared to modified cells cultivated in the absence of GXM (Figure 3c,d; *p* < 0.05). Considering the transduction at MOI 1, soluble GXM at concentrations of 1 and 0.1 μg/mL induced the activation of GXMR-8-28ζ Jurkat cells, whereas GXMR-8-BBζ promoted the highest levels of IL-2 only at 0.1 μg/mL of GXM (Figure 3c,d; *p* < 0.05). At MOI 3, both GXMR-8-BBζ- and GXMR-8-28ζ-modified cells induced an increase in IL-2 levels only at 0.1 μg/mL of GXM (Figure 3c,d). The absence of high levels of IL-2 in GXMR-IgG4-28ζ Jurkat cells incubated with soluble GXM demonstrated that the interaction between CAR and its ligand was insufficient for cell activation (Figure 3c,d). GXMR-CARs also induced the activation of modified Jurkat cells in response to distinct *Cryptococcus* species. This evaluation considered MOI 1 and MOI 3 transduced cells that were co-cultured with heat-inactivated *C. gattii* R265 or *C. neoformans* H99 yeasts, and the IL-2 levels in the cell supernatant were quantified 24 and 48 h after incubation. GXMR-8-BBζ and GXMR-8-28ζ led to the production of high levels of IL-2 after interaction with *C. gattii* or *C. neoformans* yeast compared to the modified cells in the medium alone (Figure 3e–h; *p* < 0.05). The production of IL-2 by Jurkat cells expressing GXMR-8-BBζ or GXMR-8-28ζ was significantly higher than that by GXMR-IgG4-28ζ- and Lenti-mock cells when co-cultured with *C. gattii* or *C. neoformans* yeast (Figure 3e–h; *p* < 0.05), whereas GXMR-8-BBζ induced the highest levels of IL-2 in the presence of ligands compared to all constructs (Figure 3e–h; *p* < 0.05). Interestingly, the activation of GXMR-CAR Jurkat cells induced by *Cryptococcus* yeast did not differ between strains R265 and H99 in any of the GXMR-CAR constructs studied (Figure 3e–h). The signal strength of high GXMR-8-BBζ tonic signaling increased in response to antigen recognition.

To complement the role of hinge/transmembrane and signaling domains in GXMR-CAR cells in the presence of ligands, the growth of GXMR-CAR Jurkat cells was evaluated during incubation with heat-killed *Cryptococcus* spp. Jurkat cells expressing GXMR-CAR after transduction with an MOI of one had an expansion for 48 h, as expected and already reported in Figure 2a, and the presence of strain R265 or H99 induced cell activation that resulted in reduced cell expansion (Figure 3i–k). GXMR-8-BBζ Jurkat cells showed a threefold decrease in cell concentration after interaction with *Cryptococcus* spp. yeast after 48 h of culture, whereas GXMR-8-28ζ and GXMR-IgG4 promoted four- and fivefold decreases in cell concentration, respectively (Figure 3i–k). In MOI-3-transduced Jurkat cells, the increase in GXMR-CAR expression by the augmentation of MOI and the recognition of *Cryptococcus* spp. yeast by GXMR-CARs also reduced cell expansion, mainly in GXMR-8-28ζ and GXMR-IgG4-28ζ, and the cell concentration of GXMR-8-BBζ-Jurkat cells reached 1.2- and 2.5-fold higher than GXMR-IgG4-28ζ and GXMR-8-28ζ-cell, respectively, after 48 h of co-culture with strains R265 or H99 (Figure 3l–n). Thus, the 4-1BB signaling domain favored cell activation triggered by GXMR-8-BBζ and contributed to cellular resilience during cell expansion against *Cryptococcus* spp. yeast.

### 3.5. Dasatinib, a Tyrosine Kinase Inhibitor, Attenuates the Tonic Signaling Induced by GXMR-CARs Containing CD8α as Hinge/Transmembrane

**Figure 4:**
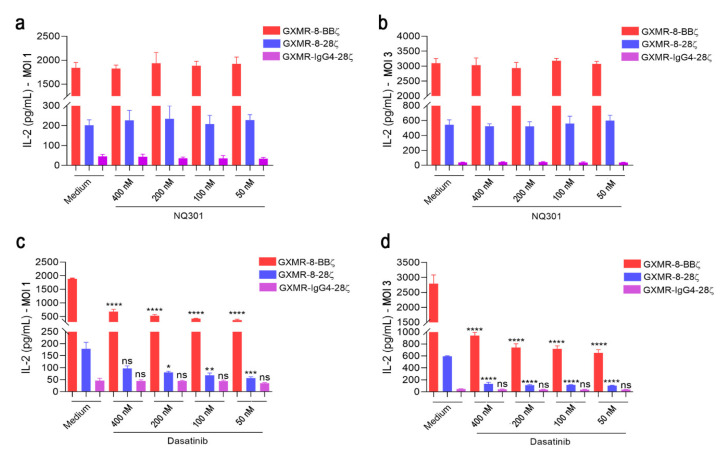
Dasatinib decreases IL-2 level induced by GXMR-CAR tonic signaling in Jurkat cells. Jurkat cells were modified by transduction with distinct GXMR-CAR constructs using an MOI of 1 or 3, and 3 days later, the modified Jurkat cells (2 × 10^5^ cells/mL) were incubated with different concentrations of NQ301 (**a**,**b**) or dasatinib (**c**,**d**). After 48 h of culture, the IL-2 levels were measured using ELISA, and as a negative control was used DMSO (Medium; a equivalent amount to the highest volume of inhibitors was added in the culture). The values are expressed as mean ± SD. Each experiment was performed in triplicate and repeated at least three times. * *p* < 0.05, ** *p* < 0.01, *** *p* < 0.001, **** *p* < 0.0001, according to one-way ANOVA. ns, not significant.

To investigate the mechanisms involved in tonic signaling triggered by specific GXMR-CAR constructs expressed in Jurkat cells, we studied the effect of CD45 phosphatase activity on the regulation of T-cell receptor (TCR) activation and the inhibition of phosphorylation motifs of CARs. The pharmacological inhibitor NQ301 blocks the tyrosine phosphatase activity of CD45, compromising signal transduction through TCR in the T-cell activation process [28]. Jurkat cells were transduced with MOI 1 and MOI 3 using distinct GXMR-CAR constructs, modified cells were treated with different concentrations of NQ301, and after 48 h of incubation the levels of IL-2 were evaluated. Inhibition of CD45 phosphatase mediated by NQ301 did not alter the levels of IL-2 produced by GXMR-CAR Jurkat cells in the absence of the ligand (Figure 4a,b). To address the CAR signaling pathway responsible for GXMR-CAR tonic signaling, an inhibitor of Src family tyrosine kinases, dasatinib, was used to block the adenosine triphosphate (ATP) binding site of the lymphocyte-specific protein tyrosine kinase (LCK) involved in CD3ζ phosphorylation [29], which has been shown to reduce cytokine production and exhaustion in CAR T cells [30]. Dasatinib at several concentrations reduced IL-2 production by GXMR-8-BBζ- and GXMR-8-28ζ-Jurkat cells that were transduced at MOI 1 and MOI 3 (Figure 4c,d), and the production of IL-2 triggered by GXMR-8-BBζ and GXMR-8-28ζ reached levels five- and fourfold lower in the presence of dasatinib (Figure 4c,d). These results indicate that the tonic signaling of the GXMR-CAR constructs containing the CD8α molecule as a hinge/transmembrane was drastically reduced when LCK-mediated phosphorylation of CD3ζ was inhibited.

### 3.6. Dasatinib Attenuates the Effects of Cell Activation Induced by GXMR-CAR Even in the Presence of Cryptococcus spp.

**Figure 5:**
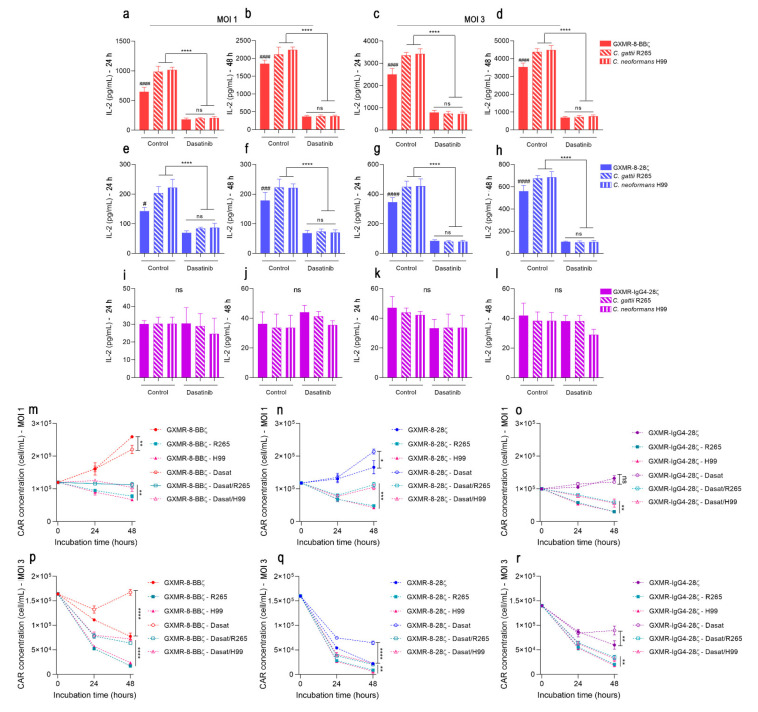
The response induced by GXMR-CAR in Jurkat cells against *Cryptococcus* spp. was ameliorated by dasatinib. GXMR-CAR constructs were used for transduction (MOI of one or three) of Jurkat cells, and 3 days after transduction, the modified cells (2 × 10^5^ cells/mL) were incubated with heat-killed yeast of *C. gattii* R265 or *C. neoformans* H99 at a ratio of 1:1 (cells:yeast) for 24 and/or 48 h of culture. This approach also considered the previous incubation with dasatinib (50 nM) before the co-culture of GXMR-CAR Jurkat cells with *Cryptococcus* spp., and as negative control, modified cells not treated with dasatinib were used. The quantification of IL-2 levels in the culture supernatant was performed after 24 and 48 h of culture (**a**–**l**), and cell expansion was measured using flow cytometry within 48 h of culture, as described in Materials and Methods section (**m**–**r**). The values are expressed as mean ± SD. Each experiment was performed in triplicate and repeated at least three times. *, # *p* < 0.05; **, ### *p* < 0.01; *** *p* < 0.001; ****, #### *p* < 0.0001, according to one-way ANOVA. ns, not significant. # denotes statistical significance comparing GXMR-CAR control without yeast against GXMR-CAR treated with dasatinib without yeast.

GXMR-CAR tonic signaling was reduced by dasatinib in Jurkat cells that were transduced with an MOI of one and three using GXMR-8-BBζ or GXMR-8-28ζ constructs. We therefore evaluated the effects of dasatinib on Jurkat cells expressing GXMR-CAR in the presence of *Cryptococcus* spp. IL-2 levels in the cell supernatant and the evaluation of cellular expansion were considered in the co-culture of GXMR-CAR Jurkat cells with heat-killed yeast for 48 h after incubation with or without 50 nM dasatinib. The high levels of IL-2 produced by GXMR-8-BBζ- and GXMR-8-28ζ-modified cells in the presence of *Cryptococcus* spp. yeast were significantly reduced to levels close to those of the negative control when dasatinib was added (Figure 5a–h). MOI 3-transduced cells expressing GXMR-8-BBζ or GXMR-8-28ζ and incubated with its ligand showed a reduction in IL-2 levels, due to dasatinib, which caused a sixfold decrease in the cytokine levels compared to the non-treated cells (Figure 5c,d,g,h). GXMR-8-BBζ and GXMR-8-28ζ Jurkat cells treated with dasatinib and incubated with or without *Cryptococcus* spp. did not differ in IL-2 levels, indicating that the inhibitory action orchestrated by dasatinib on the CAR signaling pathway is predominant even when CAR-specific ligands are available (Figure 5a–h). In contrast, the GXMR-IgG4-28ζ-carrying cells did not demonstrate tonic signaling, and IL-2 levels did not change upon CAR–ligand interaction; therefore, the cells treated with dasatinib did not show relevant changes (Figure 5i–l).

The cell expansion of GXMR-CAR Jurkat cells was also assayed in the presence of dasatinib, and GXMR-8-BBζ- and GXMR-8-28ζ-modified cells at an MOI of one and three had improved cell concentration after treatment with dasatinib compared to non-treated cells under the same conditions (Figure 5m–o). This observation was more pronounced in MOI-3-transduced cells that were treated with dasatinib. After 48 h of culture, the cell concentration was approximately 2-, 3-, and 1.6-fold higher for GXMR-8-BBζ, GXMR-8-28ζ-, and GXMR-IgG4-28ζ-Jurkat cells, respectively, compared to non-treated cells (Figure 5p,r). In addition, GXMR-CAR Jurkat cells were treated with dasatinib in the presence of *Cryptococcus* spp., and the effect on the cell concentration of GXMR-CAR Jurkat cells caused by its ligand was significantly reduced in the presence of dasatinib for all three CAR constructs (Figure 5m–r). Of note, the cell concentrations of GXMR-8-BBζ- and GXMR-8-28ζ-modified cells reached close levels after 48 h of co-culture with strains R265 or H99 in the presence of dasatinib (Figure 5m,n). Moreover, GXMR-CAR-modified cells at MOI 3 showed a significant improvement in the expansion of cells treated with dasatinib even before incubation with *Cryptococcus* spp. yeast (Figure 5p,r). Attenuation of the effect on expansion of Jurkat cells expressing GXMR-CAR in the presence of its ligand was more evident in the cells modified with GXMR-8-BBζ that were treated with dasatinib (Figure 5p). Put together, these results suggest that the signal strength of GXMR-CARs containing CD8α as a hinge/transmembrane protein promotes cell activation in an MOI-dependent manner, which can be attenuated by inhibition of the CAR signaling pathway. In addition, the co-stimulatory molecules 4-1BB and CD28 induce a distinct signal strength by GXMR-CD8-CAR in Jurkat cells, which was clearly demonstrated by the cytokine production and cellular expansion rate.

### 3.7. CD69 Expression Augmented in Jurkat Cells Modified with GXMR-CAR, after Incubation with Cryptococcus spp. Yeast, Validated a More Pronounced Signal Strength by GXMR-8-BBζ

**Figure 6:**
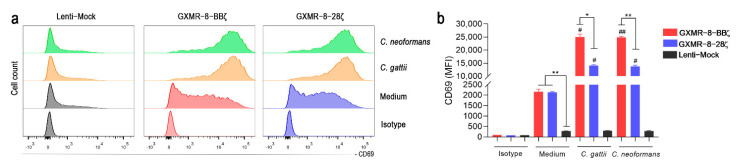
Jurkat cell activation mediated by GXMR-CAR constructs in the presence of *Cryptococcus* spp. yeasts was validated by expression of the activation marker CD69. Jurkat cells (2 × 10^5^ cells/mL) expressing GXMR-8-BBζ, GXMR-8-28ζ or Lenti-mock constructs, after transduction using an MOI of 3, were incubated with heat-killed yeast of *C. gattii* or *C. neoformans* (1:1 ratio of cells:yeast) or only medium for 20 h of culture. After this period of incubation, the cells were stained with a human anti-CD69 APC-conjugated antibody or isotype control, and the CD69 expression was measured by flow cytometry. (**a**) Representative histograms of CD69 expression and (**b**) quantification of the median of the CD69-APC fluorescence intensity after 20 h of co-culture between transduced cells and heat-killed yeast or only medium. The values are expressed as mean ± SD; and *, # *p* < 0.05, **, ## *p* < 0.01 according to one-way ANOVA followed by Tukey’s multiple comparisons test. # denotes statistical significance when GXMR-CAR constructs or Lenti-mock in the presence of heat-killed yeasts were compared to medium, whereas * represents a significant difference between GXMR-CAR and Lenti-mock in the same condition.

Jurkat cell activation is also detected by up-regulation of the CD69 expression that is an activation marker strongly associated with T cells [31]. Therefore, Jurkat cells transduced with GXMR-8-BBζ, GXMR-8-28ζ, or Lenti-mock, using an MOI of three, were incubated with heat-killed yeast of *C. gattii*, *C. neoformans*, or medium alone as a negative control. After 20 h of co-incubation, the CD69 cell-surface expression was measured by flow cytometry, and the cells modified with GXMR-8-BBζ and GXMR-8-28ζ showed a significant increase in the CD69 expression in response to yeast of *Cryptococcus* spp., compared to Lenti-mock control cells (Figure 6a,b). Moreover, the role of the co-stimulatory 4-1BB domain was noteworthy in the induction of high levels of CD69 in Jurkat expressing GXMR-8-BBζ that was more pronounced than those levels of CD69 found in cells modified with GXMR-8-28ζ, when the cells were incubated with the yeast (Figure 6a,b). In addition, the tonic signaling triggered by GXMR-8-BBζ and GXMR-8-28ζ in Jurkat cells, as reported previously in the Figure 3, did not result in a significant difference in the levels of CD69 expression between both GXMR-CAR constructs (Figure 6a,b). Therefore, alongside with these data it was corroborated that Jurkat cells can compose a platform to validate the signal strength of GXMR-CAR against the *Cryptococcus* spp. yeasts.

### 3.8. Cryptococcus spp. Clinical Isolates Are Recognized by GXMR-CARs That Mediate Cell Activation

**Figure 7:**
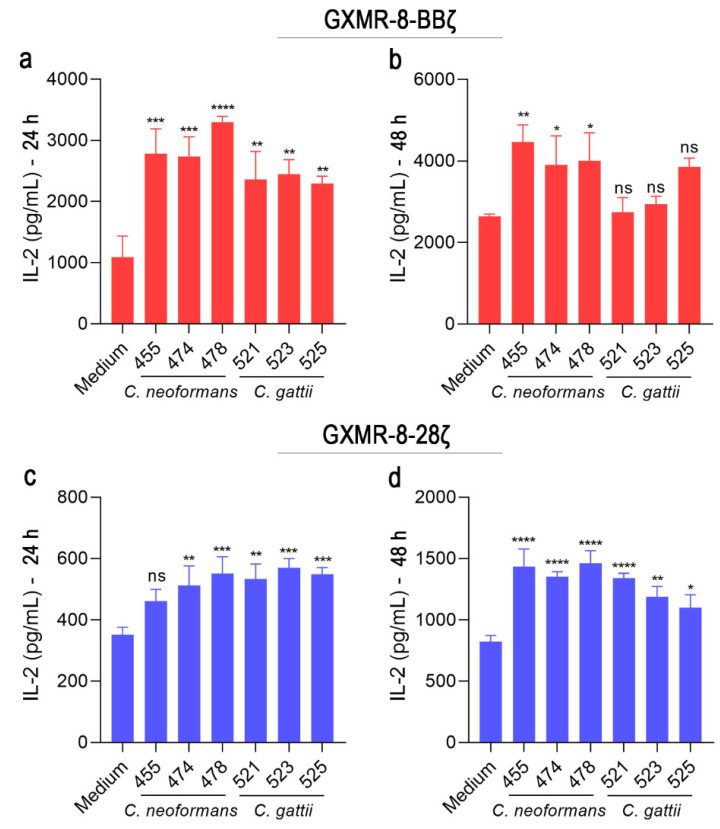
Heat-killed clinical isolates of *C. neoformans* and *C. gattii* are recognized by GXMR-CARs inducing T-cell activation. GXMR-8-BBζ and GXMR-8-28ζ constructs were used for transduction of Jurkat cells using an MOI of 3, and 3 days after transduction, the modified cells (2 × 10^5^ cells/mL) were incubated with heat-killed isolates of *C. gattii* or *C. neoformans* at a ratio of 1:1 (cells:yeast) for 48 h of culture. Shown are the quantification of IL-2 levels in the culture supernatant of GXMR-8-BBζ cells (**a**,**b**) and GXMR-8-28ζ cells (**c**,**d**). The values are expressed as mean ± SD. Each experiment was performed in triplicate and repeated three times. * *p* < 0.05, ** *p* < 0.01, *** *p* < 0.001, **** *p* < 0.0001, according to one-way ANOVA. ns, not significant.

Reproducing the findings of the study of novel chimeric receptors targeting *Cryptococcus* spp. is challenging using clinical isolates of *Cryptococcus* spp.; hence, most of the findings were assayed using reference strains. Moreover, the morphological modification of *Cryptococcus* spp. as titan cells that occur in the pulmonary tissue could be an escape mechanism used by fungi to subvert their recognition by chimeric receptors. Therefore, these factors have been studied in the context of GXMR-CAR, which has a great capacity to target *Cryptococcus* titan cells [12], and in the current study, we evaluated the ability of GXMR-8-BBζ and GXMR-8-28ζ to mediate cell activation against clinical isolates of *Cryptococcus* spp. Jurkat cells were transduced with GXMR-8-BBζ or GXMR-8-28ζ using an MOI of three, and the modified cells were incubated with heat-killed clinical isolates of *C. neoformans* or *C. gattii*. After 24 and 48 h of co-culture, IL-2 levels in the cell supernatant were measured using ELISA. GXMR-8-BBζ-modified cells showed a significant increase in IL-2 levels in all clinical isolates tested after 24 h of culture (Figure 7a), whereas IL-2 levels after 48 h were significantly augmented only in the presence of *C. neoformans* isolates (Figure 7b). GXMR-8-28ζ induced high levels of IL-2 in the presence of the clinical isolates at all periods analyzed, except for the *C. neoformans* 455 strain, specifically after 24 h of co-culture (Figure 7c,d). These results validate the great capacity of GXMR-8-BBζ and GXMR-8-28ζ to recognize *Cryptococcus* spp. that induces T-cell activation.

## 4. Discussion

T cells engineered to express chimeric antigen receptors are considered a revolution in cell and gene therapy owing to their success in the treatment of lymphocytic leukemia and promissory to treat solid tumors. The redirection of cytotoxic T cells using CAR technology to target proteins, carbohydrates, lipids, and other molecules has opened new applications of CAR technology beyond cancer [6]. Although this therapy has proven to be potent in targeting several types of epitopes, the rationale for CAR design must be considered when applied to a new ligand. Therefore, improvement of CAR T-cell function by modifying the CAR structure is desirable. CAR domains (scFv, hinge, transmembrane, and co-stimulatory portions) should be evaluated to establish efficient CAR constructs that play a pivotal role in ligand recognition, induction of cell activation, and low expression of exhaustion phenotypes. In this context, the components of GXMR-CAR, the first CAR designed to target *Cryptococcus* spp. through the scFv derived from 18B7 monoclonal antibody [24], should be investigated to evaluate the importance of hinge/transmembrane and co-stimulatory domains to improve GXMR-CAR function. In the current study, we modified the original structure of GXMR-CAR, which redirects T cells to recognize GXM in *Cryptococcus* spp. capsules, to generate new GXMR-CAR constructs containing CD8α molecules as hinge/transmembrane and CD28 or CD137 (4-1BB) as co-stimulatory domains. Therefore, the new constructs GXMR-8-28ζ and GXMR-8-BBζ had the ability to induce cell activation against *Cryptococcus* spp., and these novel GXMR-CARs showed a remarkable improvement compared to GXMR-IgG4-28ζ, which is composed of IgG4 as a hinge and CD28 as a transmembrane/co-stimulatory domain. GXMR-8-BBζ and GXMR-8-28ζ were expressed at higher levels and persisted longer during the expansion of Jurkat cells and also induced tonic signaling compared to GXMR-IgG4-28ζ. In addition, GXMR-8-BBζ and GXMR-8-28ζ increased the signal activation strength in modified Jurkat cells in response to the ligand, whereas GXMR-IgG4-28ζ did not mediate T-cell activation in the presence of the ligands. Our results showed that GXMR-CAR containing CD8α as a hinge/transmembrane combined with 4-1BB triggers a signal strength that is more pronounced in response to *Cryptococcus* spp., and Src family kinases are crucial in the GXMR-CAR signaling pathway. 

Several studies have reported that CAR expression and ligand affinity are greatly affected by scFv instability, which occurs independently of the hinge/transmembrane and signaling domains [32,33]. On the one hand, the antigen recognition domain of a CAR is commonly studied to increase its affinity and specificity to target a molecule with emphasis on the strength of the signal transmission; on the other hand, the functional strength of CAR-engineered cells can be improved by adjustment of the hinge size or by changing the molecule anchored in the cell membrane, and these portions are also correlated with CAR expression [34,35]. Considering that a smaller hinge facilitates CAR folding and expression [35], in the current study, the hinge/transmembrane domain of the original form of GXMR-CAR (containing IgG4 plus a CD28 portion, totaling 254 aa) was replaced with a CD8α molecule (71 aa), which elevated the efficiency of GXMR-CAR expression on the cell surface, maintaining antigen recognition. These findings are corroborated by a previous study that observed a higher expression of CD19-CAR when the transmembrane domain CD28 was replaced with the CD8α molecule [35]. Moreover, Jurkat cells modified with GXMR-CAR containing CD8α as the hinge/transmembrane showed a cellular expansion that was more acceptable than that observed in Jurkat cells expressing GXMR-IgG4-28ζ, and the hinge/transmembrane substitution did not impair the redirection of cells to target *Cryptococcus* spp.

The expression of GXMR-CAR stood out when CD8α molecules were inserted into the CAR constructs, which also contributed to the GXMR-CAR tonic signaling that was observed in Jurkat cells modified with GXMR-8-BBζ and GXMR-8-28ζ, whereas GXMR-IgG4-28ζ did not produce antigen-independent signaling in Jurkat cells. These observations were based on IL-2 levels and expression of the CD69 molecule by distinct GXMR-CAR T cells in the absence of *Cryptococcus* spp. Independent studies have explored the advantages of tonic signaling for antigen-independent expansion of T cells in vitro and in vivo [36,37,38,39]. The current study found that the recruitment of the tonic signaling pathway was substantially higher in GXMR-8-BBζ-modified cells, and tonic 4-1BB co-stimulation was consistently present in a variety of CAR constructs [36,37,38,39]. Moreover, GXMR-8-BBζ tonic signaling resulted in better expansion of Jurkat cells compared to cells modified with other GXMR-CAR constructs. Interestingly, the high levels of IL-2 production and CD69 up-regulation induced by GXMR-8-BBζ and GXMR-8-28ζ evidenced the tonic signaling in Jurkat cells. Moreover, the capacity of GXMR-8-BBζ and GXMR-8-28ζ to induce a response against *Cryptococcus* spp. yeast was validated by increased levels of IL-2 and CD69 up-regulation in modified Jurkat cells incubated with the ligands. Then, the evaluation of the levels of IL-2 and the CD69 expression were essential to differentiate the signal strength derived from GXMR-8-BBζ and GXMR-8-28ζ in the absence or presence of the ligands. Although GXMR-IgG4-28ζ-modified cells were able to recognize soluble GXM and heat-killed yeast, these GXMR-CARs composed of IgG4 (hinge) and CD28 (transmembrane) induced weak activation in Jurkat cells. In contrast, previous studies reported that a CAR containing IgG4/CD28 as a hinge/transmembrane was still able to mediate cell activation upon antigen binding [10].

The transduction procedure in Jurkat cells impairs their cellular expansion, as verified in Lenti-mock transduced cells, in which Jurkat cells were modified to express GFP alone, and the expression of GXMR-CAR in Jurkat cells promoted lower cellular expansion compared to Lenti-mock-modified cells. However, there was a positive effect of the 4-1BB signaling domain on the cellular expansion and activation of cells transduced with GXMR-8-BBζ, which was significantly higher than that of the other two constructs, indicating that GXMR-8-BBζ signaling prolonged cell survival. These findings were also found in the microenvironment between GXMR-8-BBζ-modified cells and *Cryptococcus* spp. and increasing GXMR-CAR expression by MOI3 or stimulating CAR engagement with its ligand augmented cell activation, which reduced cellular expansion. Several studies have demonstrated the superiority of 4-1BB signaling in ameliorating cell exhaustion or enhancing the efficacy of CAR T-cell therapy [36,40,41], although there are some concerns related to the persistence and intensity of 4-1BB co-stimulation that could up-regulate pro-apoptotic genes contributing to the apoptosis of engineered CAR T cells [39]. The current study evaluated the effect of GXMR-CAR construct expression in the presence of exhaustion markers in GXMR-CAR Jurkat cells, and there was no correlation between the increase in exhaustion marker expression and the cellular expansion rate for each GXMR-CAR construct.

The GXMR-CAR tonic signaling favored the cellular expansion mainly when modified cells were incubated with *Cryptococcus* spp.; however, this tonic signaling is commonly observed in a variety of CAR T cells and can cause adverse effects during patient treatment. Some strategies to overcome the negative effects of tonic signaling include limiting CAR expression by changing the promoter or adding an internal ribosome entry site element upstream of the LTR promoter, which is an efficient way to reduce tonic signaling [38,39]. Moreover, pharmacological agents that inhibit key molecules in the CAR signaling pathway can regulate cell proliferation, exhaustion, and apoptosis-related genes in CAR T cells, mitigating CAR-related side effects in patients. Phosphorylation by LCK protein, which is critical in the CAR signal transduction pathway, is inhibited by dasanatib [30,42,43], which can be considered to regulate the adverse side effects caused by CAR [29]. These mechanisms promoted by dasatinib were also identified in GXMR-CAR-Jurkat cells that had a significant reduction of the IL-2 production, as detected by GXMR-8-BBζ and GXMR-8-28ζ tonic signaling, as well as when the ligand was recognized. This reduction in the activation of GXMR-CAR-Jurkat cells mediated by dasatinib also recovered the cellular expansion rate of cells modified with GXMR-CAR, which was affected in response to signal strength sourced from CAR in the presence of the ligand. On the other hand, tonic signaling triggered by GXMR-CAR can induce a strong pro-inflammatory immune response in the site affected by *Cryptococcus* spp., which can be desirable mainly in the early stage of infection for controlling the progression of cryptococcosis.

The details of the GXMR-CAR structure investigated in the current study validate the capacity of variants of GXMR-CAR to recognize *Cryptococcus* spp. inducing high levels of cell activation, which will be satisfactory in the engineering of CAR T and CAR NK cells specifically to treat cryptococcosis. Further studies will explore the benefits of the signal transduction pathway of variants of the GXMR-CAR to enhance the engineered cells’ antifungal activity to subvert *Cryptococcus* species’ polysaccharide capsule, which has its composition and size rearranged in a microenvironment-dependent manner. The role of the GXM polysaccharide of *Cryptococcus* spp. is extremely relevant in the evasion of recognition by innate and adaptive immune cells and in the modulation of the host immune response favoring the progression of cryptococcosis [20]. These features are commonly studied in different *Cryptococcus* reference strains and in clinical isolates from cryptococcosis patients; therefore, this context was considered and the evaluation of activity of GXMR-CAR constructs against several clinical isolates of *Cryptococcus* spp. becomes critical. Both GXMR-8-BBζ and GXMR-8-28ζ constructs were able to mediate the activation of modified Jurkat cells after incubation with different clinical isolates from *C. gattii* and *C. neoformans,* whereas GXMR-8-BBζ presented better signal transduction to activate T cells, which reinforces the distinct potential of variants of GXMR-CAR to induce cell activation targeting GXM from the *Cryptococcus* capsule.

The characterization of distinct GXMR-CAR constructs considering the modifications in the hinge/transmembrane and signaling transduction domains was performed in the Jurkat cell line, in which the laborious isolation, expansion, and transduction of primary T cells for CAR expression can be circumvented using Jurkat cells. This strategy, reported by Bloemberg et al., aimed to evaluate the sensitivity and specificity of several antigen-binding domains composed of CARs, and tonic signaling and target-specific activation mediated by CAR were measured using a high-throughput method in Jurkat cells [44]. Assessing CAR activation in Jurkat cells does not predict the whole effect triggered by the signal transduction pathway of CAR that this molecule could induce in primary T cells due to its differentiation and effector function characteristics. Otherwise, Jurkat cells expressing CAR could indeed predict tonic signaling, ligand-specific activation, and cell surface expression of CAR in primary human T cells [44], which optimizes the development of novel CARs based on time effectiveness and cost efficiency when Jurkat cells are used.

In conclusion, this study demonstrated the optimization of novel second-generation GXMR-CARs by changing the hinge, transmembrane, and co-stimulatory domains that improved CAR expression, antigen recognition, and signal strength in T-cell activation. These optimized GXMR-CARs will allow further studies to emphasize the engineering of primary T cells to express GXMR-CAR in the context of in vitro and in vivo *Cryptococcus* spp. infection. The characterization of novel GXMR-CARs in Jurkat cells opens new opportunities for further development of CARs for cell therapy against invasive fungal infections.
